# Conjunctival myxoma: A case report and review of a rare tumor

**DOI:** 10.1097/MD.0000000000037342

**Published:** 2024-03-08

**Authors:** Seong Eun Lee, Sung Bok Lee, Kyunghee Kim, Jae Yun Sung

**Affiliations:** a Department of Ophthalmology, Chungbuk National University Hospital, Cheongju, Republic of Korea; b Department of Ophthalmology, Chungnam National University College of Medicine, Chungnam National University Hospital, Daejeon, Republic of Korea; c Department of Pathology, Chungnam National University College of Medicine, Chungnam National University Hospital, Daejeon, Republic of Korea; d Department of Ophthalmology, Chungnam National University College of Medicine, Chungnam National University Sejong Hospital, Sejong, Republic of Korea.

**Keywords:** conjunctival benign tumor, conjunctival cyst, conjunctival myxoma

## Abstract

**Rationale:**

Conjunctival myxoma is a rare benign tumor, which can mimic more common conjunctival lesions such as a cyst, lymphangioma, amelanotic nevus, neurofibroma, amelanotic melanoma, or lipoma. We describe a patient with the conjunctival myxoma, who was initially misdiagnosed as a conjunctival cyst. This case report includes intraoperative photographs and various immunohistochemical staining images.

**Patients concerns:**

A 55-year-old woman presented with a painless mass in the superotemporal conjunctiva of the left eye, which she had noticed 1 month ago. The patient had no previous history of trauma or eye surgery. Slit-lamp examination revealed a well-circumscribed, freely movable, pinkish, semi-translucent mass on the temporal bulbar conjunctiva, suggestive of a conjunctival cyst.

**Diagnoses:**

Histopathological analysis showed stellate- and spindle-shaped cells within the loose myxoid stroma, confirming a diagnosis of conjunctival myxoma.

**Interventions:**

The conjunctival lesion was completely excised under local anesthesia.

**Outcomes:**

After 4 months of follow-up, the patient remained in good health without recurrence of the conjunctival lesion and no evidence of any systemic abnormality.

**Lessons:**

Myxoma is an extremely uncommon benign tumor derived from primitive mesenchyme. Considering the rarity of the tumor and its similarity to other conjunctival tumors, diagnosis can be challenging. Ophthalmologists should consider myxoma as a possible differential diagnosis when encountering conjunctival lesions. Surgical excision is essential to confirm the diagnosis and careful systemic evaluation is required to prevent potentially life-threatening underlying systemic conditions.

## 1. Introduction

A myxoma is a rare benign tumor derived from primitive mesenchyme that can manifest in various locations including the heart, bone, skin, skeletal muscle, and nasal sinuses, as well as the genitourinary and gastrointestinal systems.^[1,2]^ Ocular myxomas are extremely rare, but have been documented in the orbit, eyelid, cornea, conjunctiva, and lacrimal gland.^[3–10]^ The tumor may be isolated or constitute a component of the Carney complex, that includes cutaneous or cardiac myxomas with pigmented skin or ocular lesions, and endocrine overactivity.^[11]^

In a clinical survey of 1643 patients with conjunctival lesions, myxoma was only identified in 1 (0.06%).^[12]^ Considering the rarity of the tumor, a conjunctival myxoma can simulate or mimic several more common conjunctival lesions such as a cyst, lymphangioma, amelanotic nevus, neurofibroma, amelanotic melanoma, or lipoma.^[7,9]^ Typically, myxomas present as slow-growing, painless, fleshy gelatinous masses, and appear to be pink or yellow in color.^[13]^ If such a lesion is present, a myxoma should be considered in the differential diagnosis, and along with surgical excision, histopathological analysis must be performed to establish an accurate diagnosis.

Here, we describe a patient with the typical clinical presentation and histopathological characteristics of a conjunctival myxoma, who was initially misdiagnosed as a conjunctival cyst. Intraoperative photographs and images of various immunohistochemical stains are included in this case report.

## 2. Case report

A 55-year-old woman presented with a painless mass in the superotemporal conjunctiva of the left eye, that she had noticed 1 month prior. She did not report any discomfort; she sought medical consultation for cosmetic reasons. There was no other systemic disease except for dyslipidemia. The patient had no previous history of trauma or eye surgery.

**Figure 1.**
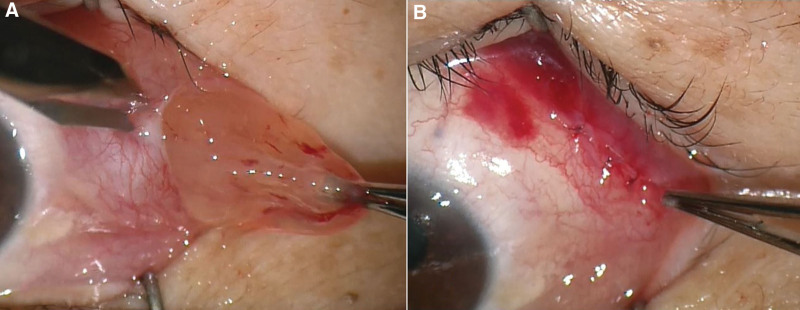
(A) Clinical appearance of a conjunctival myxoma of the left eye. Slit-lamp image showing a well-circumscribed, pinkish, semi-translucent mass on the bulbar conjunctiva, suggestive of a conjunctival cyst. (B) Intraoperative image captured during excisional biopsy under local anesthesia. After conjunctival incision, the mass exhibited a fleshy gelatinous texture, with no adhesion to surrounding tissues, and there was minimal bleeding due to the scarcity of blood vessels.

The best corrected visual acuity was 20/20, and intraocular pressures were normal in both eyes. During external examination, an elevated mass was observed in the superotemporal quadrant of the left eye; this protruded more noticeably when the patients looked downward. Slit-lamp examination of the left eye revealed a well-circumscribed, freely movable, pinkish, semi-translucent mass on the temporal bulbar conjunctiva (Fig. 1A). The corneas, anterior chambers and fundus examinations of both eyes were unremarkable. The initial impression was a conjunctival cyst. The lesion was completely excised under local anesthesia. Grossly, the mass exhibited a fleshy and gelatinous appearance, unlike a cyst; it was measured 1.3 × 1.0 mm in size. The mass showed no adhesion to surrounding tissues and bleeding was minimal because of blood vessel scarcity in the region (Fig. 1B). Ofloxacin eyedrops and ointment were prescribed for 1 week postoperatively.

**Figure 2.**
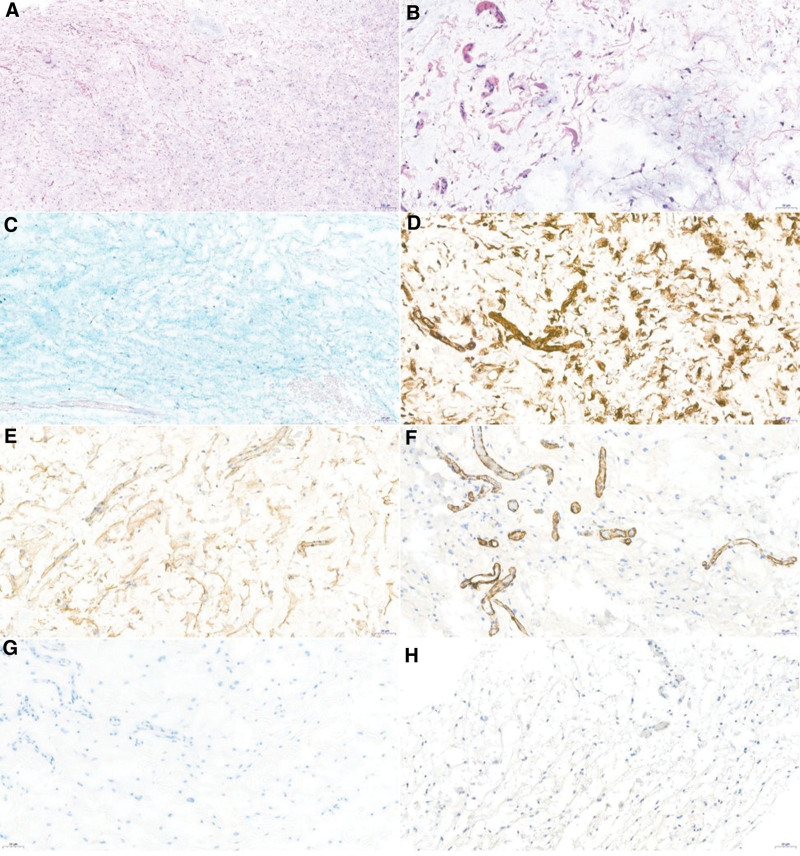
Histology of the conjunctival myxoma. Spindle- and stellate-shaped cells dispersed in a loose myxoid stroma. (A) Hematoxylin and eosin stain, ×50 (scale bar: 50μm). (B) Hematoxylin and eosin stain, ×200. (C) Mucoid material was confirmed by positive staining with Alcian blue (×200). Immunohistochemical staining results were positive for (D) Vimentin (×200) and (E) CD34 (×200) but negative for (F) α-SMA (×200), (G) S-100 (×200), and (H) STAT6 (×200). α-SMA = alpha smooth muscle actin.

Histopathological examination revealed stellate- and spindle-shaped cells within the myxoid stroma, as well as scattered vascular tissues. Mucoid material stained positive for Alcian blue. Immunohistochemical staining revealed that the tumor was positive for vimentin and CD 34 but negative for alpha-smooth muscle actin (α-SMA), S-100, and STAT6 (Fig. 2). These features were consistent with a diagnosis of conjunctival myxoma. The possibility of Carney complex and Zollinger-Ellison syndrome was considered. Systemic evaluations of cardiac myxoma, endocrine abnormalities and cutaneous pigmentation showed negative results. After 4 months of follow-up, the patient remained in good health without recurrence of the conjunctival lesion and no evidence of any systemic abnormality.

## 3. Discussion

Conjunctival myxomas are exceedingly uncommon; the incidence is 0.06% to 0.16% of all conjunctival tumors. In a histopathological review of 2455 conjunctival lesions in adults, Grossniklaus et al only identified 4 myxomas.^[14]^ In a clinical survey of 1643 patients with conjunctival lesions, only 1 myxoma was found.^[12]^ Myxomas generally occur in older adults; they are rare in younger populations.^[6]^ In a literature review of 45 studies including 78 patients, the mean patient age was 45.1 ± 16.6 years (ranging, 11 months to 80 years); only 5 patients (6.4%) were aged < 18 years.^[15]^ Our patient was slightly older than average. No gender preference was found in previous studies,^[9,16,17]^ however, in a recent review, male individuals were more frequently affected than female individuals (ratio 6:4).^[13]^ Conjunctival myxomas usually are not associated with a history of trauma or surgery, as in our patient. However, some patients have reported histories of foreign body injuries in the affected eye and prior surgery at the lesional sites, including cataract,^[18]^ strabismus,^[19]^ and epiblepharon^[16]^ surgery.

Typically, a conjunctival myxoma presents as a painless, slow-growing, movable, translucent, yellow-pinkish conjunctival mass. However, diagnosis can be challenging for ophthalmologist due to its rarity and its similarity to other tumors in the conjunctiva. The most common initial impression is cyst (30.8%), followed by a tumor (10.2%), lymphoproliferative disorder (8.9%), and dermolipoma (7.7%). Our patient was initially misdiagnosed with a conjunctival cyst.^[15]^ Conjunctival myxomas tend to develop on the bulbar conjunctiva, followed by the limbal, fornix, and palpebral areas.^[17]^ They are more frequent on the temporal rather than the nasal side,^[8,17]^ which aligns with the presentation in our patient.

Grossly, specimens are most commonly described as soft, gelatinous, and fleshy.^[15]^ In our case, similarly, the cut surface of the specimens was white-to-yellow, gelatinous, and not encapsulated. Histopathologically, conjunctival myxomas echibit of stellate- and spindle-shaped cells embedded in a hyaluronic acid-rich mucinous matrix, accompanied by sparse vascular and fibrillary structures,^[7]^ consistent with our finding. The review article by Alvarado-Villacorta et al^[15]^ reported that all immunohistochemically evaluated conjunctival myxomas showed extensive reactivity with vimentin (19/19, 100%), and most cases demonstrated strong and diffuse immunoreactivity for CD34 (18/20, 90%). Eighty-eight percent of cases were negative for S-100 (37/42) and α-SMA (22/25). No lesions demonstrated immunoreactivity for SRY-Box Transcription Factor 10 or desmin. The findings in our case were similar. The myxoma cells revealed positive for vimentin and CD34, but negative for S-100 and α-SMA.

The differential diagnosis of conjunctival myxoma includes amelanotic nevus, amelanotic melanoma, fibrous histiocytoma, cyst, myxoid neurofibroma, lymphangioma, spindle cell lipoma, rhabdomyosarcoma and myxoid liposarcoma.^[7,9,17,20]^ Conjunctival amelanotic nevi and melanomas are both characterized by prominent intrinsic vascularity or pigmentation; myxomas lack such features.^[9,14,20]^ Fibrous histiocytomas are distinguished from myxomas by the vascularity and significant nuclear pleomorphism.^[9]^ Conjunctival cysts are lined with layers of non-keratinized epithelium and connective tissue, that are filled with clear serous fluid, whereas myxomas do not exhibit these layers.^[21]^ Myxoid neurofibromas and myxomas initially appear to be similar; however, Myxoid neurofibromas are not cystic, and they exhibit spindle-shaped nuclei and wavy collagen bundles that are often associated with systemic neurofibromatosis.^[7,9]^ Lymphangiomas are highly vascularized, whereas myxomas are not.^[17]^ Spindle cell lipomas tends to be more yellowish and they contain mature fat cells and signet ring cells compared to myxoma.^[20,22]^ Rhabdomyosarcomas typically occurs in childhood or adolescence, presenting as small, round cells mixed with stellate cells that exhibit mitotic activities; myxomas lack these cells.^[23]^ In contrast to liposarcomas, myxomas lack pleomorphic multivacuolated lipoblasts and signet ring cells.^[20]^

The recommended treatment of conjunctival myxoma is complete excision. Recurrence after excision is rare. Recurrence was reported in only 1 case, which occurred 12 months after surgery in association with Carney complex.^[11]^ Recurrence of myxoma may reflect inadequate resection, tumor multicentricity, or a genetic predisposition to recurrence.^[17]^ No malignant transformation of a conjunctival myxoma has been reported, although some cardiac myxomas proceed to malignancy.^[20,24]^

At presentation, our patient had no systemic comorbidity except for dyslipidemia; however, it is important to note that some myxomas may be a component of the Carney complex^[11]^ or may be associated with Zollinger–Ellison syndrome or an abnormal atrial septum thickness.^[25]^ Carney complex is an autosomal dominant syndrome that meets at least 2 of the following criteria: myxoma (cardiac, cutaneous, or mammary), spotty mucocutaneous pigmentation (of the face, trunk, lips, eyelid, or conjunctiva), endocrine overactivity (including Cushing syndrome), pituitary adenoma (associated with acromegaly or gigantism) and/or psammomatous melanotic schwannoma.^[26]^ The ophthalmic manifestations of Carney complex include facial and eyelid lentigines, caruncle or conjunctiva pigmentation, and eyelid myxomas.^[17,27]^ Considering the potential associations, it is imperative to conduct a comprehensive systemic evaluation, which should include cardiac sonography, screening for endocrine overactivity, examination of skin or ocular pigmentation, and a thorough assessment of the medical history of the patient relatives.

In conclusion, we present the case of a 55-year-old woman with a conjunctival myxoma, a rare, benign conjunctival tumor. The clinical and histopathological features were in good agreement with descriptions of this rare tumor in the literature. Considering the rarity of the tumor and its similarity to other conjunctival tumors, diagnosis can be challenging. Ophthalmologists should consider myxoma as a possible differential diagnosis when encountering conjunctival lesions. Surgical excision is essential to confirm the diagnosis; careful systemic evaluation is required to prevent potentially life-threatening underlying systemic conditions.

## Author contributions

**Conceptualization:** Seong Eun Lee, Jae Yun Sung.

**Data curation:** Seong Eun Lee, Jae Yun Sung.

**Formal analysis:** Seong Eun Lee, Kyunghee Kim, Jae Yun Sung.

**Investigation:** Seong Eun Lee, Sung Bok Lee, Kyunghee Kim, Jae Yun Sung.

**Methodology:** Sung Bok Lee, Jae Yun Sung.

**Supervision:** Sung Bok Lee, Jae Yun Sung.
